# An updated view on lagging strand DNA replication: implications for the replication stress response

**DOI:** 10.1080/15384101.2026.2646889

**Published:** 2026-03-22

**Authors:** Rodrigo Martín-Rufo, Emilio Lecona

**Affiliations:** Department of Genome Dynamics and Function, Centro de Biologia Molecular Severo Ochoa (CBM), CSIC-UAM, Madrid, Spain

**Keywords:** DNA replication, lagging strand, DNA polymerase alpha/primase, replication stress response, Okazaki fragments, VCP/p97

## Abstract

The process of DNA replication is inherently asymmetric. While the leading strand is synthesized continuously, the lagging strand is copied in small fragments, the Okazaki fragments, requiring the repeated priming by the DNA polymerase alpha/Primase complex (Pol α/Pri). Current evidence is consistent with a semi-distributive model for priming in the lagging strand, as Pol α/Pri acts associated to the replisome and also as a free complex. In addition, there is a strong link between the dynamics of replication in the lagging strand and the basal activation of the replication stress response (RSR) during an unperturbed S phase. We hypothesize that the RSR monitors the generation of Okazaki fragments to control the synthesis of DNA in what we call the DNA replication control (DRC) mode of the RSR. The DRC enforces a gradual progression of DNA replication by restricting origin firing, what is necessary to establish the replication program in the cell and to prevent the appearance of genomic instability. Thus, the RSR coordinates the replication program in the cell, modulating the progression of DNA replication to prevent the exhaustion of cellular resources that would endanger the stability of the genome.

## 1. Introduction

The cell cycle is the ordered sequence of events that enables, first, the duplication of the genetic information and cellular organelles, and then, their distribution between the two daughter cells in mitosis [1]. DNA replication lies at the heart of the cell cycle: it has to be faithful and accurate to maintain genomic stability, requiring a tight and strict coordination that prevents the acquisition of mutations and the onset of disease. Even if the duplication of the DNA takes place in S phase, the process of DNA replication spans the whole interphase. During the G1 phase, cells accumulate the resources to duplicate their DNA and mark the regions of the genome where DNA replication will start in S phase in a process known as origin licensing. The success of DNA replication relies on the ordered copy of different regions of the genome, as established in the replication program. This program is set in G1 through the licensing of origins of replication and executed by the progressive firing of a limited number of these origins in S phase. At the end of S phase most of the genome has been replicated and cells progress into a transition step, the G2 phase. In G2 the cells solve the problems that arose during DNA replication and complete the full copy of the genome before starting the mitotic program.

Copying over three billion base pairs in each S phase is an enormous challenge. To preserve the integrity of the genetic information, the cell counts with the replication stress response (RSR), a specialized pathway activated by problems arising through DNA replication. The RSR stabilizes stalled replication forks and activates specific DNA damage response pathways. If the damage is persistent or too high, the RSR activates the G2/M checkpoint that prevents the progression into mitosis in the presence of damaged and/or unreplicated DNA [2]. Of note, this pathway is essential even during an unperturbed S phase. While the activation of the RSR by exogenous damage and replication stress (RS) has been extensively studied, we still do not fully understand how cells set the basal activation of the RSR in an unperturbed S phase and why it is essential. Cancer cells often display increased levels of RS, making them more dependent on the basal activation of the RSR. Here, we review the recent evidence which puts forward lagging strand replication as a sensor of DNA replication. We propose a semi-distributive model for Okazaki fragment generation that is linked to the basal activation of the RSR. We hypothesize that, in unperturbed conditions, the RSR works in a DNA replication control (DRC) mode, that is driven by the dynamics of DNA replication in the lagging strand.

## 2. The asymmetry of DNA replication

The DNA replication program begins in G1 through the licensing of the origins of replication. The loading of the ORC (Origin recognition complex) in specific regions in the genome leads to the recruitment of CDC6 and CDT1. Together, they promote the incorporation of two copies of the hexameric minichromosome maintenance (MCM2-7) complex, constituting the pre-replication complex (pre-RC) [3]. Only a fraction of the pre-RC is going to be activated, and the rest of licensed origins remain in a “dormant” state, ready to be fired in the face of problems that hinder the progression of DNA replication [4,5]. The activation of the pre-RC, or origin firing, consists in a two-step process, triggering the initiation of DNA replication and the transition from G1 to S. First, the phosphorylation of the pre-RC by the Dbf4-dependent kinase (DDK) and S phase CDK promotes the loading of Cdc45, GINS, and DNA polymerase ε to form a double CMGE complex. Then, MCM10 induces the separation of each CMGE, opening the dsDNA to allow the loading of the rest of the replication machinery and the formation of a bidirectional DNA replication fork [3,6–9]. In this process, the CMG extrudes the lagging strand and surrounds the leading strand, establishing the asymmetric structure of the forks, that is essential to allow the synthesis of the DNA by replicative DNA polymerases [10].

**Figure 1.**
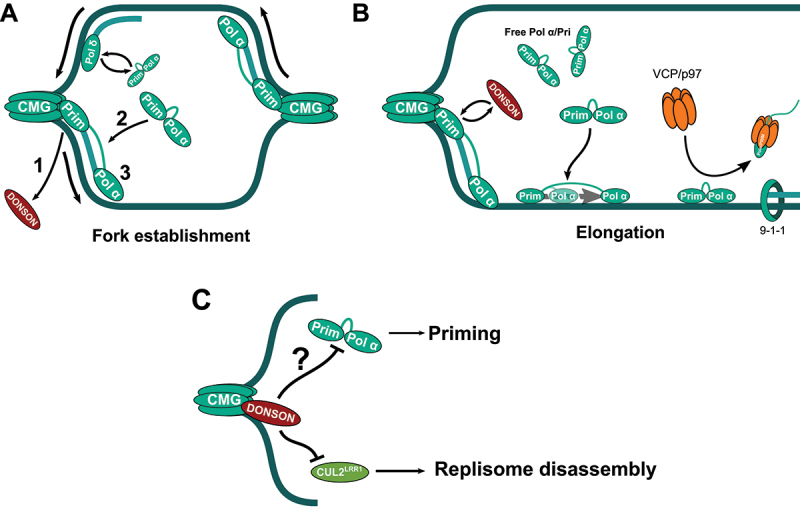
Semi-distributive model for priming during lagging strand DNA replication. (A) Upon origin firing, Pol α/Pri replaces DONSON in the CMG, setting the initial primer in both the leading and the lagging strand. (B) During fork elongation, Pol α/Pri primes the lagging strand, working in association with the CMG or from the free pool in the nucleus. VCP/p97 limits priming by extracting Pol α/Pri from chromatin. (C) Pol α/Pri, DONSON and CUL2^LRR1^ compete for the same binding surface in the CMG complex, what could be relevant to control the priming in the lagging strand and to prevent replisome disassembly.

Three polymerases from the B family, DNA polymerase α, δ and ε, mediate the copy of the DNA in S phase. Neither Pol δ nor Pol ε can initiate DNA synthesis by themselves, as they require the presence of a primer annealed to ssDNA to add dNTPs at the 3’ end. Thus, DNA synthesis is initiated by the DNA polymerase α/Primase complex (Pol α/Pri) through the synthesis of RNA-DNA primers on the ssDNA that has been exposed in both strands upon origin firing (Figure 1(A)). Then, these primers are elongated by Pol δ until this polymerase catches up with the CMG in the leading strand, when Pol δ is replaced by CMG-coupled Pol ε. The synthesis of the lagging strand requires the repeated priming by Pol α/Pri and the extension of these primers by Pol δ to synthesize the Okazaki fragments [11,12] (Figure 1(B)). The continuous synthesis of the leading strand and the discontinuous synthesis of the lagging strand are thus enforced by the directionality of DNA synthesis by replicative DNA polymerases, acting in the 5’-3’ direction. The synthesis of DNA by Pol δ and Pol ε is supported by the Proliferating Cell Nuclear Antigen (PCNA) complex that enhances the processivity of DNA polymerases [3,13]. A close interaction between PCNA and Pol δ in the presence of ssDNA explains the preferred use of this polymerase in the lagging strand [12,14]. Once Pol δ reaches the next Okazaki fragment, it displaces the RNA primer creating a 5’ flap structure that is cleaved by the endonuclease FEN1. Alternatively, a longer flap can be generated by displacing both the RNA and the DNA parts of the primer. In this case, the DNA2 nuclease first removes part of the flap to allow the subsequent action of FEN1 or the exonuclease EXO1. Last, the DNA nick is ligated by DNA ligase I, leading to a continuous lagging strand [15].

## 3. DNA polymerase α/Primase: priming and beyond

### 3.1. Priming during DNA replication

Pol α/Pri is a hetero-tetrameric complex with two functional modules: the DNA primase, composed by the catalytic subunit PRIM1 and the regulatory subunit PRIM2; and the DNA polymerase module, formed by POLA1, the catalytic subunit, and POLA2, with regulatory roles [11,16]. In the apo state, PRIM1 scans for a template while the catalytic domain of POLA1 remains blocked thanks to its interaction with other components of the complex. Upon ssDNA binding, PRIM1 performs the synthesis of an 8–10 nucleotides long RNA primer, what triggers a conformational rearrangement that releases POLA1 and positions it at the tip of the RNA primer. Then, POLA1 takes over to extend the RNA primer, generating an RNA-DNA primer of approximately 25 nucleotides in length. PRIM2 remains anchored to the 5’ end of the nascent RNA-DNA primer during the elongation by POLA1, imposing a spatial constraint that limits primer length [11,16].

Pol α/Pri synthesizes these RNA-DNA primers while establishing several direct contacts with the CMG helicase in the replisome. PRIM2 binds both MCM3 and the GINS complex, and the interaction with GINS contributes to the priming in the lagging strand [17]. The direct binding to the CMG positions Pol α/Pri right at the site where the lagging strand is extruded by the helicase, while the leading strand remains far away from Pol α/Pri, encircled by the CMG [17]. In this configuration, Pol α/Pri can directly overcome the competition with the RPA complex for the binding to ssDNA and mediate priming in the lagging strand. This configuration would also explain why the action of Pol α/Pri is restricted to this strand [17]. In the presence of damage, Pol α/Pri contributes to DNA damage tolerance by re-priming in the lagging strand to allow DNA replication restart when the replication fork is stalled [18,19]. In the leading strand, on the other hand, it is the primase/polymerase PRIMPOL who is in charge of re-priming to resume DNA synthesis [20–22].

### 3.2. Priming-independent functions for Pol α/Pri

The interaction of Pol α/Pri with specific factors in the replisome drives priming-independent functions of the complex during DNA replication. The AND1/Ctf4 trimer serves as a binding platform that provides multiple contacts with different replication factors, including Pol α/Pri [23,24]. However, the interaction of Pol α/Pri with AND1/Ctf4 is not essential in yeast [25], and the loss of this interaction does not affect priming or the replication of the lagging strand in reconstituted systems [26]. Instead, AND1/Ctf4 promotes the chaperone activity of Pol α/Pri during chromatin re-assembly, facilitating the transfer of parental histones to the lagging strand [25,27]. In addition, Pol α/Pri is recruited to telomeres by the CST (CTC1-STN1-TEN1) complex [28]. CST binds telomeric ssDNA and recruits Pol α/Pri [29], that is kept in its apo inactive state by POT1 [30]. A conformational switch ensues, leading to the positioning of the primase and polymerase modules in Pol α/Pri to start the synthesis of the C-strand in the telomere [31]. The CST-Pol α/Pri complex also participates in the repair of DNA double-strand breaks where it carries out fill-in activities [32].

The multiple functions of Pol α/Pri during DNA replication are shaped by its interaction with diverse factors at the replisome. The high abundance of this complex in the nucleus suggests that Pol α/Pri can dynamically associate with these replication factors to control and coordinate priming, histone chaperoning and fill-in activities during DNA replication. Still, priming is the essential function of Pol α/Pri. Different models have been proposed to explain how the action of Pol α/Pri is controlled during the generation of the RNA-DNA primers.

## 4. Priming in the lagging strand: processive or distributive?

Structural evidence and reconstituted DNA replication systems support a processive model for priming in the lagging strand where Pol α/Pri stays attached to the replisome while the fork progresses. *In vitro*, disrupting the interaction of Pol α/Pri with the CMG helicase leads to longer and less abundant Okazaki fragments, despite not completely abolishing priming. However, introducing these mutations does not affect viability in yeast [33], challenging the model for a completely processive priming of Pol α/Pri coupled to the progress of the replication forks. During DNA replication, Pol α/Pri would need to synthesize the primer in the lagging strand while still in contact with the CMG helicase, which unwinds the parental DNA in the opposite direction. Since PRIM2 stays attached to the 5’ end of the primer and also mediates the interaction with the CMG, it is unclear if both contacts can be maintained when priming happens. Alternatively, it is possible that Pol α/Pri detaches from the replisome when it starts its priming activity.

### 4.1. DONSON comes into play

The recent discovery of the replication initiation factor DONSON in higher eukaryotes poses new questions regarding the association of Pol α/Pri with the replication machinery. During origin licensing, DONSON is recruited by the action of DDK and S phase CDK to the pre-RC, where it has been proposed to act as the functional homolog of yeast Sld2 [34–39]. In the pre-RC, DONSON interacts with TOPBP1 and the MCM complex to promote the incorporation of GINS and the formation of the CMG complex [36–38]. Furthermore, DONSON has been suggested to promote the loading of CDC45 and Pol ε, although these functions seem to be species specific [40]. A recent report shows that RECQL4 induces the exit of DONSON from the replisome right before origin firing is completed [35]. Interestingly, the docking site for DONSON in the CMG overlaps with the interaction site for Pol α/Pri [17,37], as both of them contact the same region in MCM3 [17,40]. These results suggest that DONSON needs to be removed for Pol α/Pri to access the replisome and carry out the initial priming in both strands after origin firing (Figure 1(A)). However, DONSON is dispensable for the progression of replication forks [40,41] and reconstituted human replisomes lacking DONSON sustain DNA replication at normal rates [26]. Even if it is not required for DNA replication elongation, DONSON prevents the premature ubiquitylation and disassembly of the replication machinery by blocking the access of the E3 ubiquitin ligase CUL2^LRR1^ to its substrate, the MCM complex [42]. Thus, the association of DONSON to the replication machinery would be necessary to prevent the premature disassembly of the replisome. Again, this argues against a stable association of Pol α/Pri with the CMG during the elongation of active replicating forks.

### 4.2. The regulated association of Pol α/Pri with the lagging strand

Together, the current evidence suggests an interplay between Pol α/Pri and DONSON during DNA replication competing for the same binding sites in the CMG. An on/off interaction of Pol α/Pri with the replisome is consistent with a semi-distributive model for priming in the lagging strand. The Smith lab carried out sequential depletion experiments to analyze the effect of gradually reducing Pol α/Pri in Okazaki fragment generation in yeast [33]. In a processive model, a partial reduction in the levels of the complex should not affect priming, as a small pool of Pol α/Pri stably tethered to the CMG would be sufficient to support DNA replication in the lagging strand. In contrast, depleting Pol α/Pri first increases the size of Okazaki fragments, and only when the amount of Pol α/Pri is strongly reduced, a defect in origin firing is also observed [33]. Further, the loss of the interaction of Pol α/Pri with Ctf4/AND1 is only relevant for priming when the amount of Pol α/Pri is limiting [33]. Similar to the data in yeast, *in vitro* experiments using the bacterial DNA replication machinery show that the amount of primase directly determines the abundance of Okazaki fragments independent of the progression of the DNA polymerases in the leading strand [43]. Together, these data point toward a semi-distributive model of priming, with a pool of free Pol α/Pri contributing to priming in the lagging strand during fork progression, and a second pool of Pol α/Pri bound to the CMG and with an essential role in origin firing.

The semi-distributive model for priming posits the following question: how is the action of the free pool of Pol α/Pri limited to prevent generating too many Okazaki fragments? We have recently shown that Pol α/Pri is removed from chromatin by VCP/p97, an AAA ATPase that targets ubiquitylated and SUMOylated proteins through a number of different cofactors [44]. In chromatin, VCP/p97 unfolds and removes proteins that are no longer needed on the DNA, leading to their degradation or recycling [45]. VCP/p97 is involved in the extraction of replication factors in S phase, maintaining the ubiquitin and SUMO balance at replication forks in cooperation with the USP7 deubiquitylase [46]. Furthermore, VCP/p97 extracts the replication machinery after DNA replication termination [47–50]. Our data show that the inhibition of VCP/p97 also leads to the accumulation of Pol α/Pri on chromatin and an increase in the abundance of Okazaki fragments (Figure 1(B)) [44]. In addition, it is possible that the extraction of Pol α/Pri by VCP/p97 plays a role in the polymerase exchange in the lagging strand, being a necessary step to load Pol δ.

### 4.3. A semi-distributive model for priming in the lagging strand

Based on these data, the current view for the replication of the lagging strand involves the semi-distributive generation of Okazaki fragments thanks to the regulated priming by Pol α/Pri (Figure 1(B)). The tethering of Pol α/Pri to the CMG during origin firing would be required to establish bidirectional replication forks (Figure 1(A)). During DNA replication fork progression, priming would be carried out by the free pool of Pol α/Pri, and its presence on chromatin would be controlled by VCP/p97 (Figure 1(B)). A pool of Pol α/Pri associated with the replisome could also contribute to priming during DNA replication elongation or could be necessary for re-priming in the lagging strand, maybe in difficult to replicate regions. It is not clear how the binding of Pol α/Pri to the CMG is affected by the competition with DONSON and CUL2^LRR1^ during the progression of the fork, as well as in response to fork stalling (Figure 1(C)). Since these proteins play an important role in S/G2 transition and in replisome disassembly after DNA replication termination, it is possible that the association of Pol α/Pri with the replisome and the priming in the lagging strand are regulated throughout the S phase.

The dynamics of the replication of the lagging strand impact the duplication of the DNA. Increasing evidence links lagging strand replication with the basal activation of the RSR, the main pathway controlling the fitness of DNA replication and the stability of the genome.

## 5. The replication stress response

### 5.1. Mechanisms of activation of the RSR

The progression of DNA replication forks can be hampered by a wide variety of challenges: a depletion in the pool of deoxynucleotides (dNTPs), the presence of inter-strand crosslinks (ICLs), G-quadruplexes, DNA lesions and R-loops, among others [2]. The RSR works as an alarm that senses these problems, activating specific pathways to protect stalled replication forks and promote DNA replication restart and repair. In addition, the RSR activates the G2/M checkpoint to avoid the progression to mitosis in the presence of unreplicated or damaged DNA [2]. The main transducer in the RSR is the kinase ATR, a member of the PI3K-like family. Its activation leads to the phosphorylation of different substrates, including the Checkpoint Kinase 1 (CHK1), the main effector of the cellular responses of the RSR [51–53].

**Figure 2.**
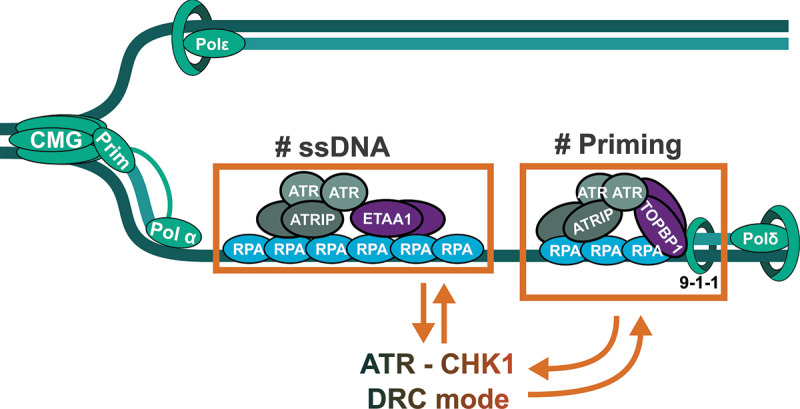
Lagging strand replication drives the activation of the RSR. Schematic representation of lagging strand replication, showing how the generation of ssDNA and primed DNA structures set the basal activity of ATR/CHK1, the DNA replication control mode, DRC. While ETAA1 binds to ssDNA, TOPBP1 is loaded in primed DNA structures by the 9-1-1 complex. Conversely, the DRC regulates the dynamics of DNA replication, establishing a reciprocal control between DNA replication and the RSR.

When the progression of replication forks is hampered, the CMG helicase and the DNA polymerases uncouple, exposing ssDNA. Given the fragile nature of ssDNA, it is rapidly coated by the RPA complex to protect it from degradation. At the same time, RPA serves as a platform to recruit the ATR-ATRIP (ATR-interacting protein) complex through a direct binding of ATRIP to RPA [53]. To achieve its full activation, ATR requires the stimulation from specific co-activators, mainly TOPBP1 and ETAA1 (Figure 2) [54–57]. TOPBP1 recruitment is achieved when, in response to RS, the 9-1-1 complex (RAD9-HUS1-RAD1) is loaded on the free 5’ end in ssDNA-dsDNA junctions, enabling the activation of the adjacent ATR. In contrast, ETAA1-mediated activation of ATR only requires RPA-coated ssDNA, as ETAA1 also interacts with the RPA complex [54]. Both TOPBP1 or ETAA1 have been shown to form homodimers that contribute to the activation of ATR [58]. The phosphorylation of CLASPIN by ATR constitutes the docking point for inactive CHK1. ATR-mediated phosphorylation of CHK1 leads to its activation and release from chromatin [53].

### 5.2. Effects of the RSR

The RSR displays a slew of responses to the presence of stalled DNA replication forks. It stimulates the synthesis of deoxynucleotides (dNTPs) by increasing the levels of ribonucleotide reductase to support DNA replication [59–61]. Further, it protects stalled replication forks through, at least, four different actions. First, the RSR induces the phosphorylation of SMARCAL1 to limit the extent of fork regression [62]. Second, it promotes the recruitment of homologous recombination (HR) factors and FANCD2 to protect the regressed forks from degradation by nucleases [63,64]. Third, it prevents the premature activation of G2/M specific nucleases such as SLX4, that would lead to fork degradation and collapse [65]. Last, the RSR stimulates the action of the BLM and WRN helicases, required for the recovery of stalled forks [66,67]. In parallel, the RSR activates several pathways to deal with the damage that causes the fork stalling, including HR and the translesion synthesis pathways, as part the of the DNA damage tolerance response [68]. In addition to promoting fork restart [53], the RSR also activates repriming mechanisms by PRIMPOL in the leading strand and by Pol α/Pri in the lagging strand [18,69–71]. Together, the RSR protects both ongoing and nascent replication forks [72] and, at the same time, promotes the progression of DNA replication.

**Figure 3.**
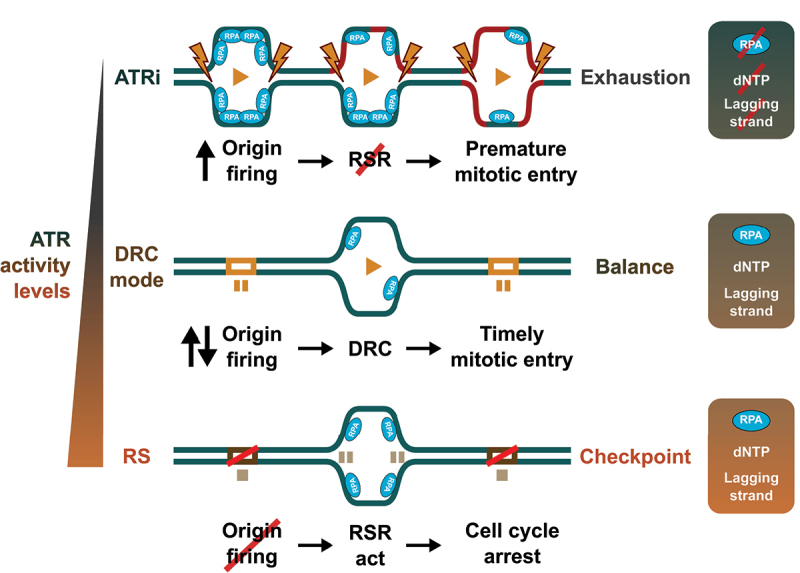
ATR activity and the control of DNA replication. Schematic representation of the regulation of origin firing by different levels of ATR activity. In unperturbed conditions (middle, balance), the DRC mode of the RSR enforces the progressive firing of origins of replication preventing the exhaustion of DNA replication factors and substrates, and allowing the timely activation of the mitotic program. The inhibition of ATR (top, exhaustion) leads to the premature activation of origins, an excessive amount of DNA replication and the exhaustion of the pool of dNTPs, RPA or of lagging strand replication factors and, at the same time, pushing cells prematurely into mitosis. An increase in ATR activity (bottom, checkpoint) activates the checkpoint and induces the inhibition of origin firing, preventing the progression of S phase in the presence of damage.

In addition to the direct regulation of the DNA replication machinery, the RSR modulates the cell cycle program and the activation of the G2/M checkpoint. In the presence of high levels of RS, CHK1 activates the G2/M checkpoint and delays the entry into mitosis [2]. To avoid the onset of genomic instability, the sustained activation of the checkpoint induces apoptosis or senescence [53]. To activate the checkpoint, CHK1 phosphorylates the CDC25 phosphatase, inducing its degradation and resulting in the inhibition of CDK1 and CDK2. The reduced activity of CDK also impacts DNA replication dynamics as it prevents the assembly of the active CMG helicase and inhibits origin firing. By limiting the firing of new origins, the reduced CDK activity prevents the exposure of additional ssDNA that could exhaust the RPA pool in the cell and induce the so-called replication catastrophe [73]. However, low levels of RS need to be compatible with the firing of dormant origins to ensure the completion of DNA replication [4,74]. In this sense, origin firing is directly modulated by the RSR through the control of FANCI phosphorylation [75]. When the activation of the RSR is weak, FANCI favors the firing of dormant origins. Increasing RS leads to the phosphorylation of FANCI by ATR, favoring its association with FANCD2 to promote the recombination-based repair of replication forks and inhibiting the firing of new origins [75]. Thus, the activation of the RSR gradually restricts origin firing, allowing a slow firing of dormant origins in the presence of low levels of stress, while completely blocking origin firing in response to high levels of RS (Figure 3).

### 5.3. The RSR in cancer cells

This homeostatic control of origin firing is disrupted during malignant transformation. In this context, the functions of the RSR described in normal cells, now become essential for cancer cell survival. Oncogene activation is associated with increased RS and DNA damage, along with the activation of the DNA damage response (DDR), in what is known as the oncogene-induced DNA damage model [76]. The overexpression or mutation of oncogenes, such as Myc, Ras and Cyclin E, leads to the unscheduled firing of origins of replication that increases RS in cancer cells [2,77]. The subsequent activation of the RSR acts as a mechanism to prevent cellular transformation [2]. Reducing RS by increasing the supply of nucleotides [78] or by increasing the amount of CHK1 [79] promotes cellular transformation as it limits the damage induced by oncogene activation. Interestingly, the activation of the RSR by oncogenes is also linked to the induction of genomic instability and tolerance to RS. The expression of mutant Ras induces tolerance to RS by promoting ATR activity [80,81]. Cyclin E overexpression, instead, induces whole-genome duplication upon activation of the RSR [82]. Once they have overcome the initial barrier for malignant transformation, cancer cells rely on a high basal activity of the RSR to grow in the presence of persistent RS. Thus, the RSR becomes essential to maintain the fitness of tumor cells and inhibiting ATR or CHK1 limits tumor growth, making them a promising target for cancer treatment [2]. Both ATR and CHK1 inhibitors show potent antitumor effects either alone or in combination with traditional chemotherapy and DNA damaging agents, and are currently undergoing clinical trials [83].

Thus, we have a comprehensive picture of how the activation of the RSR is elicited in response to RS, and how the RSR deals with the problems induced either by external agents or by pathological situations, such as the process of malignant transformation. Of note, the deletion of ATR or CHK1 is lethal at the organism and cellular level even in unperturbed conditions [84–86]. This suggests that the RSR is not only an alarm activated by the presence of RS but it is also critical for the progression of DNA replication.

## 6. The regulation of unperturbed DNA replication by the RSR

The loss of the RSR machinery is not compatible with cell survival and the depletion or inactivation of the RSR leads to the development of Seckel and other related syndromes [87–90]. A mouse model of the Seckel syndrome, mimicking the defect in the splicing of ATR observed in patients, shows that a limited activity of the RSR impairs cell proliferation in unchallenged conditions [91]. In particular, the loss of ATR exhausts the stem cell compartment in different tissues and results in the premature aging of the mice. Interestingly, Seckel mice do not develop tumors upon oncogene activation or the loss of tumor suppressor genes, similar to the observations performed with ATR and CHK1 inhibitors [91,92]. All these genetic evidence supports a role for the RSR in the control of DNA replication, different from the response to RS. In this sense, phosphoproteomic studies revealed that ATR targets different sets of proteins during an unperturbed S phase than in the presence of external challenges that induce RS [93]. Further, work in yeast revealed a basal level of ATR activation during DNA replication that is comparable with the effect of exposure to RS [94]. Thus, we distinguish two modes of action for ATR/CHK1. In the absence of damage, the RSR works in a DNA replication control mode (DRC) to ensure the accurate duplication of the genome. This DRC mode is associated to the basal levels of activity of ATR and CHK1 in S phase, being the essential function of the RSR pathway. In the presence of RS, the activation of ATR/CHK1 is linked to DNA repair and checkpoint activation.

In the DRC mode, ATR and CHK1 restrain CDK activity. In fact, CDK2 activity during an unperturbed S phase correlates with the basal activity of ATR [95]. By restraining CDK1 and CDK2, ATR/CHK1 controls fork progression and origin firing, setting DNA replication dynamics during S phase. This is supported by two observations. First, the depletion of CHK1 results in the inactivation of Polη due to its phosphorylation by CDK. The reduction in the translesion synthesis activity of Polη leads to the accumulation of replication fork barriers that result in a slowdown of replication forks [84]. Second, the inhibition of CDK2 by the RSR limits the firing of origins of replication during an unperturbed S phase [95–97]. In addition, the reduction in CDK1 activity by the basal activity of ATR prevents the phosphorylation of the RIF1-PP1 complex, leading to its stabilization. In turn, the RIF1-PP1 phosphatase counteracts the action of DDK and CDK2 and limits the firing of new origins [98]. Limiting origin firing seems to be key in the control of DNA replication by the DRC of ATR/CHK1. An increase in the firing of new origins is observed upon ATR inhibition, leading to increased bulk DNA replication [2,86,95,99], the subsequent exhaustion of the RPA and dNTP pools, and the collapse of replication forks [2,73,84] (Figure 3). A similar effect is observed upon the depletion of ATR in lymphocytes, that leads to replication catastrophe in early S phase due to the exhaustion of the dNTP pools. Inhibiting new origin firing rescues the effect of the loss of ATR in S phase [85]. All these data suggest that the DRC mode of action of the RSR during DNA replication prevents the excessive firing of origins in S phase (Figure 3).

Thus, the basic function of the RSR is to ensure a gradual progression of the replication of the genome in S phase. In the DRC state, the RSR activity restrains the firing of origins, making sure that the cell does not exceed its replicative capacity to prevent fork collapse and the generation of DNA damage (Figure 3) [100,101]. The progressive activation of origins can be linked to the establishment of the replication timing of different regions in the genome [102]. It has been shown that the different regions of the genome present specific times for their replication, according to their chromatin status in each cell type [102]. Euchromatin is preferentially replicated in early S phase, while heterochromatin is copied in late S phase, giving rise to a replication program that is important to maintain cell identity through cell division [103,104]. Since the DRC mode of the RSR prevents the premature activation of origins, it could contribute to set the correct replication timing program. Interestingly, it has been proposed that the coactivators of ATR, TOPBP1 and ETAA1, act in different stages of the S phase, with ETAA1 playing a more prominent role in late S phase to control the progression into mitosis [105]. In late S phase, ETAA1 mediated ATR activation prevents the phosphorylation of FOXM1 by CDK1, that is necessary for the induction of the mitotic transcriptional program [106], directly connecting the progression of DNA replication with the activation of mitosis. Thus, it is possible that the DRC state of the RSR is sequentially controlled by TOPBP1 and ETAA1 to regulate DNA replication at different stages of S phase. In addition, the DRC mode of the RSR and lagging strand DNA replication dynamics could contribute to determine cell identity through the regulation of chromatin reestablishment after DNA replication. Then, the question arises as to how the RSR monitors the progression of DNA replication to establish the proper progression of S phase.

## 7. DNA replication control of ATR activity

### 7.1. ssDNA and primed DNA drive ATR activation

Work in yeast and using *Xenopus* egg extracts has shown that ATR activation is elicited by the process of DNA replication itself [94,107]. Two factors drive ATR activation: the generation of ssDNA and the presence of primed ssDNA structures with a free 5’ end [108]. ssDNA is not only necessary for the direct recruitment of the ATR-ATRIP complex but it also contributes to ATR activation through several complementary pathways. First, the coactivator ETAA1 directly binds RPA bound to ssDNA (Figure 2). Second, RPA interacts with the ssDNA binding protein ZNF827, which also contributes to the recruitment of ETAA1 and TOPBP1 [109]. Third, RPA generates a hub that promotes the local crowding and activation of ATR-ATRIP [110]. During an unperturbed S phase, the accumulation of RPA on chromatin is limited by its interaction with SUMOylated HNRNPA2B1 that reduces the availability of the soluble RPA complex [111]. Upon RS, HNRNPA2B1 is deSUMOylated and the crowding of ATR-ATRIP is favored through the increased number of contacts with RPA [110]. In addition, both TOPBP1 and the base excision repair factor APE1 have been shown to promote the formation of condensates that enhance ATR activity [112,113]. In turn, the phosphorylation of the RPA complex by ATR leads to its extraction, limiting RPA accumulation [110]. In line with the hypothesis proposed by Moiseeva and Bakkenist [114], ATR crowding could distinguish two modes of ATR activation: the DRC, associated with low crowding of ATR, and the activation of the RSR driven by high crowding of ATR induced by ssDNA accumulation.

On the other hand, the primed ssDNA structures mostly correspond to the primers generated by Pol α/Pri, which promote the loading of the 9-1-1 clamp in the 5’ end of the primer, leading to the recruitment of TOPBP1 (Figure 2) [94,108,115–118]. Additional experiments confirmed that RSR activation can be triggered even with only the RNA part of the primer synthesized by Pol α/Pri [119]. Our lab recently described that priming by Pol α/Pri controls the basal activation of ATR during an unperturbed DNA replication [44]. We observed that Pol α/Pri is extracted from chromatin by the VCP/p97 AAA ATPase, limiting the priming in the lagging strand and preventing the excessive activation of ATR. When VCP/p97 is inhibited, the accumulation of primers leads to the activation of the RSR in a TOPBP1-dependent manner. Furthermore, the activation of the RSR induced by VCP/p97 inhibition can be prevented by inhibiting origin firing [44]. As a result, the RSR activates the checkpoint and arrests cells in G2/M after the treatment with VCP/p97 inhibitors. Interestingly, the partial inhibition of Pol α/Pri activates the RSR, due to the accumulation of ssDNA [44,120]. Similar to the accumulation of ssDNA, the primed ssDNA contributes to the control of DNA replication by ATR or to the activation of the RSR, depending on the abundance of Okazaki fragments (Figure 2).

### 7.2. Lagging strand replication is the driver of ATR activity

Both ssDNA and primed DNA are produced during the generation of Okazaki fragments in the lagging strand (Figure 2). We propose that ATR gauges the dynamics of DNA replication in the lagging strand, setting ATR/CHK1 activity in the DRC mode. This system would allow cells to “count” Okazaki fragments to measure the extent of DNA synthesis that is taking place at any given point in S phase. At the same time, the levels of ssDNA would be a sensor of the presence of problems that slowdown DNA replication. In fact, impairing the maturation of Okazaki fragments results in the activation of ATR [15,121]. Recent work from the Diffley lab further supports the connection between lagging strand replication and the RSR. In budding yeast, uncoupling the CMG from the progression of the DNA polymerases does not prevent the priming action of Pol α/Pri in the lagging strand, similar to what was observed using a bacterial DNA replication model in vitro [43]. Under these conditions, the activation of the RSR is required to prevent the accumulation of incomplete Okazaki fragments that sequester key DNA replication factors [122]. The slowdown of DNA replication by the RSR prevents nascent strand degradation and the depletion of replication factors that are necessary for DNA synthesis restart [122]. Similarly, excessive priming in human cells leads to the sequestration of key replication factors, limiting fork restart, leaving unprotected Okazaki fragments susceptible to the degradation by the HLTF nuclease, and leading to DNA replication fork collapse [123]. These results suggest that Okazaki fragments are sensed by the RSR to prevent the exhaustion of replication factors that would be the result of an excessive amount of active DNA replication forks. In this sense, PAF15 has been recently identified as a limiting factor for the replication of the lagging strand [124]. PAF15 associates to PCNA only in the lagging strand and ensures the correct maturation of the Okazaki fragments. When ATR is inhibited, the increase in origin firing leads to a higher number of active forks but the limiting amounts of PAF15 cannot cope with this increased demand, leading to impaired Okazaki fragment maturation [124]. It is tempting to speculate that PAF15 acts as a probe to measure the abundance of active forks. When deregulated, PAF15 unbalance will result in problems in the lagging strand and the activation of the RSR. Based on all these data, we propose that the DRC mode of ATR/CHK1 monitors the replication in the lagging strand to control the progression of DNA replication enabling the gradual firing of origins during S phase (Figure 3).

## 8. Perspectives

To achieve the full duplication of the DNA and maintain the stability of the genome, DNA replication needs to proceed in an ordered manner that is established by the replication program of the cell. This is executed by the gradual firing of origins of replication in different regions of the genome. Recent advances have put forward the role of the RSR in the control of unperturbed DNA replication through the regulated firing of origins of replication. In what we call the DRC (DNA replication control) mode, the RSR is activated by the process of DNA replication itself, in the absence of damage or stress. In particular, the replication of the lagging strand controls the activation of ATR, that is determined by the number of Okazaki fragments and the accumulation of ssDNA.

DNA replication has been shown to act as a brake for cell cycle progression. It has been postulated that entry into mitosis is prevented by the presence of active replication forks, to ensure the completion of DNA duplication [125]. Our model implies that the DRC activation of the RSR would be gradually reduced along S phase, allowing for the progressive activation of CDK1. We have previously described that USP7 connects S phase control and CDK1 activation, in this case through the regulation of the protein phosphatase PP2A [46,101,126,127]. Interestingly, the concomitant inhibition of DNA replication initiation and CHK1 also leads to the activation of mitotic kinases [125,128]. How these pathways are integrated to enforce the G2/M checkpoint remains to be studied.

The dynamics of DNA replication change during S phase and so does the activation of the RSR. In fact, the coactivators of ATR present stage-specific functions, which suggests that the DRC mode of the RSR could also be regulated through S phase. It is not known if the changes in the dynamics of DNA replication can be reflected in a differential activation of ATR that could be important to support the duplication of regions of the genome with specific chromatin structures. In this sense, a recent report has shown that the replication of centromeric regions is associated with a specific composition of the DNA replication machinery that influences the activation of ATR [129]. In the future, we will gain a better understanding on the interplay between ATR, the DNA replication dynamics and chromatin structure.

Finally, the machinery involved in lagging strand DNA replication is involved in the resistance to ATR and CHK1 inhibitors. Clinical data, genome-wide screenings and cell biology experiments suggest that the fitness of Okazaki fragment synthesis and maturation is a key factor in the toxic effect of RSR inhibitors in cancer [130–132]. Further research will help elucidate how we can use the connection between lagging strand replication and the RSR to treat cancer or to promote healthy aging.
